# Supplementary material for: Rapid turnover of life-cycle-related genes in the brown algae
Source: Genome Biol. 2019 Feb 14;20:35. doi: 10.1186/s13059-019-1630-6 (PMC6374913; doi:10.1186/s13059-019-1630-6)
Supplement: Supplementary file 3 — Protein alignments, corresponding Gblocks html file and CDS sequences for the lineage conserved gametophyte-biased genes. (ZIP 2109 kb) [file 13059_2019_1630_MOESM3_ESM.zip › Con_GA_alignments/Ec-01_009090.aa_ali.fasta-gb.htm]

Ec-01\_009090.aa.fasta.trans.aa\_ali.fasta


## Gblocks 0.91b Results

Processed file: **Ec-01\_009090.aa.fasta.trans.aa\_ali.fasta**  
Number of sequences: **2**  
Alignment assumed to be: **Protein**  
New number of positions: **397** (selected positions are underlined in blue)

```
                         10        20        30        40        50        60
                 =========+=========+=========+=========+=========+=========+
Ec-01_009090     MSLRVARRKLRVQWADEAEGIPISDPLKSVGHDHDDHAAVMSARCNLSEEGKEDDSDEGD
MSTRG.23221.1    ------------------------------------------------------------
                                                                             


                         70        80        90       100       110       120
                 =========+=========+=========+=========+=========+=========+
Ec-01_009090     HETWFPGKRVSRTRQYSSCILRQALPTTSPIKLRHCPQTSNCNYGEADEEQGFRDDNNST
MSTRG.23221.1    ------------------------------------------------------------
                                                                             


                        130       140       150       160       170       180
                 =========+=========+=========+=========+=========+=========+
Ec-01_009090     HSYTSESTRSGTCNSDEEPESKRENIWGGDTGATIMVERGQHGDDGDIALVAAPVHASSP
MSTRG.23221.1    ------------------------------------------------------------
                                                                             


                        190       200       210       220       230       240
                 =========+=========+=========+=========+=========+=========+
Ec-01_009090     LLSLPGPPGPRSREQAGKASSSSTVMIRDHTFSVSPQARDSAPLSLSPQPVAVFRSRNSN
MSTRG.23221.1    ------------------------------------------------------------
                                                                             


                        250       260       270       280       290       300
                 =========+=========+=========+=========+=========+=========+
Ec-01_009090     YTRSSQHKHSSTAIHEPRARCGCRGLTVRIDSGPLGLSLEASYRVGETIVLKQAWSDCAA
MSTRG.23221.1    ------------------------------------------------------------
                                                                             


                        310       320       330       340       350       360
                 =========+=========+=========+=========+=========+=========+
Ec-01_009090     DSGMFAHSMLVSNFPGKVDDDQVRVLDLGLDGRSSSSGLIKVRAVGLERNHATTTVHTRQ
MSTRG.23221.1    ------------------------------------------------------------
                                                                             


                        370       380       390       400       410       420
                 =========+=========+=========+=========+=========+=========+
Ec-01_009090     GSMLNGAPSPPNESRGSVVGVLELDEGDILVRVDNVQVTKKSFTEVASLLDQAERPTRLT
MSTRG.23221.1    ------------------------------------------------------------
                                                                             


                        430       440       450       460       470       480
                 =========+=========+=========+=========+=========+=========+
Ec-01_009090     FVRHACGRRFVGRDNGYGGGGTCRGAARHKPPCRVAYCGGWDGCCGMMGKSGCGGKQPPN
MSTRG.23221.1    ------------------------------------------------------------
                                                                             


                        490       500       510       520       530       540
                 =========+=========+=========+=========+=========+=========+
Ec-01_009090     ASCVGRVVSSPSPKIETEADGCPVAVILRARRQLRSGGMEQWHELVASRGHLWQRLLRNL
MSTRG.23221.1    --------------------------------------MEQWHEVVASRGQLWQKLLKVL
                                                       ######################


                        550       560       570       580       590       600
                 =========+=========+=========+=========+=========+=========+
Ec-01_009090     SPDSHSDRAKLRKLVGCSRSVDEHALNGRTGLVKRKLRNNYQRLISSRKGARKGSNTDGC
MSTRG.23221.1    SPDSHSDREKLRKLTGCARMLDGHPLHGKTGISKRKLTNNFQRLINGRKAVRNGGGADGS
                 ############################################################


                        610       620       630       640       650       660
                 =========+=========+=========+=========+=========+=========+
Ec-01_009090     NKAKRALAARVWKDVNRTMSPRRHGTRPGFFSASQSGGGGHVCPAHFAGGAKHAVFRVAF
MSTRG.23221.1    AQIIRALSARVWKDVNRAMSPRRPGERPGFFSASQHGGGGHVCPAHFEGGSKDAVFRVAY
                 ############################################################


                        670       680       690       700       710       720
                 =========+=========+=========+=========+=========+=========+
Ec-01_009090     ATASTLQFVSGISYMAALVLVQEPLEAMAYATLVTLLQERVMQALFPLGDVDLLDAYHLC
MSTRG.23221.1    ATASTLQFVSGISYMAALVLVQEPAEAMAYATLVTLLQEQVMQALFPLGDVDLLDAYHLS
                 ############################################################


                        730       740       750       760       770       780
                 =========+=========+=========+=========+=========+=========+
Ec-01_009090     GGRVVTFRAFGAARSPTPSEPAARGKSGDGQYP-FRGQGHRRMAEGDTRSKVVSAGSPRE
MSTRG.23221.1    GGKVVTFRAFGAARSPTSCEAAASDEDCDGQYQSSRDHGNRCTAGGEVSADGVSVGLAYD
                 ################################   #####################    


                        790       800       810       820       830       840
                 =========+=========+=========+=========+=========+=========+
Ec-01_009090     DKATTIRDTGGADRACGWQMGELEEEPTRQVVVGEMLPRLVRIFGKALKRRLPALHGHLC
MSTRG.23221.1    NVDAVGCDGDDGDSAAGRGMGKRVEEPTQQVVVGDMLPRLVRMFGKALKRRLPALHSHLC
                        #####################################################


                        850       860       870       880       890       900
                 =========+=========+=========+=========+=========+=========+
Ec-01_009090     KEAIPMSFLCAEWFTTAYARNTPLPLALCALDLFLVRLDDVMIRLGLAILEVLAPSLKML
MSTRG.23221.1    KQAIPMSFLCAEWFTTAYARNTPLPLALCAFDLFLVRLDDVMLRLGLAILEVLAPSLKAL
                 ############################################################


                        910       920       930       940       950       960
                 =========+=========+=========+=========+=========+=========+
Ec-01_009090     NGLEILLQYGNLTANVSFQEVLRCALATQSTRVSMGATKISTTLLLPERMTTWSPSRGRR
MSTRG.23221.1    NGLEILLQYGGLAANVCFEEVLRCALATQVRIN------------------------GPD
                 #############################                               


                 
                 ======
Ec-01_009090     FRGMYA
MSTRG.23221.1    ATGLSK
```

```
Parameters used
Minimum Number Of Sequences For A Conserved Position: 2
Minimum Number Of Sequences For A Flanking Position: 2
Maximum Number Of Contiguous Nonconserved Positions: 8
Minimum Length Of A Block: 10
Allowed Gap Positions: None
Use Similarity Matrices: Yes
```

```
Flank positions of the 3 selected block(s)
Flanks: [519  752]  [756  776]  [788  929]  

New number of positions in Ec-01_009090.aa.fasta.trans.aa_ali.fasta-gb:  397  (41% of the original 966 positions)
```
